# Pervasive gene deregulation underlies adaptation and maladaptation in trimethoprim-resistant *E. coli*

**DOI:** 10.1128/mbio.02119-23

**Published:** 2023-11-30

**Authors:** Rhea Vinchhi, Chetna Yelpure, Manasvi Balachandran, Nishad Matange

**Affiliations:** 1 Department of Biology, Indian Institute of Science Education and Research, Pashan, Pune, India; Yale School of Medicine, New Haven, Connecticut, USA

**Keywords:** antimicrobial resistance, two-component signaling, negative feedback, gene regulatory networks, adaptive laboratory evolution

## Abstract

The archetypal PhoQP two-component system from Enterobacteria regulates pathways like magnesium homeostasis in *Escherichia coli* and virulence factor expression in *Salmonella enterica*. We had previously reported that *E. coli* rapidly accumulated mutations in the *mgrB* gene, a negative feedback regulator of PhoQP, when evolved in the antibiotic trimethoprim. Here, we first show that trimethoprim-selected mutations in *mgrB* either lower its expression or alter the C-terminus of the MgrB protein and prevent interaction with PhoQ. Both mechanisms compromise MgrB activity, leading to PhoQP hyperactivation and overexpression of dihydrofolate reductase (*folA*), which is the target of trimethoprim. We then investigate the consequences of deregulating PhoQP for the fitness of *E. coli* and elucidate the underlying mechanisms. Using laboratory evolution, we demonstrate that *mgrB* mutations facilitate rapid fixation of resistant bacteria in populations evolving in trimethoprim, even though their independent effect on drug IC50 is nominal. This effect is explained by a pervasive transcriptional response to deregulated PhoQP, specifically on the downstream RstA-regulon, in addition to activating *folA* transcription. Pervasive gene deregulation also explained the fitness costs of *mgrB* mutations, although involving different molecular players. PhoQP hyperactivation perturbed the balance of RpoS- and RpoD-regulated transcriptional programs and mutations that reset this balance-restored bacterial fitness in antibiotic-free conditions. Our study shows that the deregulation of a single signaling pathway permeates the wider gene expression network and leads to adaptation or maladaptation depending on the environmental context. The implications of our findings for the evolution of feedback mechanisms in two-component signaling are discussed.

**IMPORTANCE:**

Bacteria employ a number of mechanisms to adapt to antibiotics. Mutations in transcriptional regulators alter the expression levels of genes that can change the susceptibility of bacteria to antibiotics. Two-component signaling proteins are a major class of signaling molecule used by bacteria to regulate transcription. In previous work, we found that mutations in MgrB, a feedback regulator of the PhoQP two-component system, conferred trimethoprim tolerance to *Escherichia coli*. Here, we elucidate how mutations in MgrB have a domino-like effect on the gene regulatory network of *E. coli*. As a result, pervasive perturbation of gene regulation ensues. Depending on the environmental context, this pervasive deregulation is either adaptive or maladaptive. Our study sheds light on how deregulation of gene expression can be beneficial for bacteria when challenged with antibiotics, and why regulators like MgrB may have evolved in the first place.

## INTRODUCTION

Signal transduction pathways perform the vital function of altering cellular physiology in response to the environment. Signaling cascades culminate in gene expression states that dictate the behavior of cells and facilitate adaptation to stressful environments. In most cellular organisms, signaling proteins are organized into gene regulatory networks. Network organization not only brings in robustness and exquisite control of gene expression but also allows for cross talk between signaling cascades, ultimately shaping which genes are expressed and to what extent (1, 2).

Two-component systems are a major class of signaling proteins used by bacteria (3–5). The first component, referred to as “sensor kinase,” auto-phosphorylates at a conserved histidine residue upon activation. It then transfers its phosphate group to a conserved aspartate residue on a second protein, called the “response regulator,” which is usually a transcription factor itself. Phosphorylation status of the response regulator directly alters its ability to bind to gene promoters and modify expression (3, 5). Several variations on this basic schema are observed in nature, including dual kinase-phosphatase activity of the sensor kinase (6), multiple phosphorelay steps (3, 7), and crosstalk between non-cognate kinases and response regulators (8, 9). Two-component systems across bacteria orchestrate responses to a wide range of cues such as metal ions (10), pH (11), nutrients (12), antimicrobial peptides (13, 14), quorum-sensing molecules (15), and cell envelope stress (16, 17). More recently, two-component sensor kinases have been proposed as novel targets for the design of antimicrobials owing to their association with drug resistance in several clinically relevant bacterial species (18–20).

The PhoQP two-component system is a prototypical signaling pathway consisting of the membrane-anchored sensor kinase, PhoQ, and cytosolic response regulator, PhoP (11). The activity of this two-component system is modulated by environmental cues such as pH, cationic peptides (14, 21, 22), and Mg^2+^ ions (11, 23). The PhoQP pathway was initially characterized as a sensor for Mg^2+^ as it is inhibited by high concentrations of extracellular Mg^2+^, and active PhoP enhances the expression of the MgtA Mg-transporter (11, 23, 24). In low Mg^2+^, PhoP also transcriptionally up-regulates other effectors of magnesium homeostasis such as the small regulatory RNA MgrR and the small polypeptide MgtS which enhance the expression of the phosphate and cation symporter, PitA (25). PhoQP has since been associated with the regulation of several other gene functions in *E. coli* and related species, most notably, expression of virulence factors in many animal and plant bacterial pathogens (26–28). Likewise, biofilm formation in *Salmonella enterica* is also modulated by the activity of PhoQP (29). Given the role of PhoQP as a major regulator of bacterial stress response, it is a potential target to inhibit bacterial pathogenesis (20, 30).

A ubiquitous feature of gene regulatory networks, seen from bacteria to humans, is the presence of feedback (1, 2, 31–34). Positive feedback or feed-forward loops in regulatory networks ensure rapid “switch-like” behavior in response to an activating signal (34, 35). Negative feedback on the other hand dampens, restricts, or resets activation levels of a signaling network. Negative feedback is thought to be corrective and is required to calibrate levels of gene expression and buffer noise (36–38). The PhoQP system in *E. coli* and related bacteria shows both kinds of feedback. PhoP binds to the *phoQP* promoter and transcriptionally activates its own expression, setting up a feed-forward loop (39). PhoP also activates the expression of a small protein called MgrB, which inserts into the bacterial inner membrane, binds to PhoQ, and inhibits it. Thus, MgrB serves as a negative feedback regulator of PhoQP signaling (40). Theoretical and empirical work have demonstrated that these nested positive and negative feedback loops can modulate the amplitude and kinetics of PhoQP signaling and ultimately shape bacterial physiology (41, 42).

We and others have shown that mutations in the *mgrB* gene are rapidly enriched in *E. coli* bacteria that were exposed to the antibiotic trimethoprim (43, 44). Mutations in MgrB resulted in PhoQP-dependent overproduction of dihydrofolate reductase (DHFR), the target of trimethoprim, which conferred antibiotic tolerance (44, 45). Interestingly, mutations in *mgrB* in clinical strains of *Klebsiella pneumoniae* and *Enterobacter* sp. are causally linked to colistin resistance and heteroresistance (21, 46), demonstrating that the deregulation of PhoQP can be beneficial in different bacterial species under antibiotic pressure. In this study, we build on our earlier observations to investigate how crucial mutational loss of negative feedback in PhoQP is for the evolution of trimethoprim resistance in *E. coli*, and why negative feedback may have evolved and is maintained by two-component signaling pathways.

## RESULTS

### Antibiotics select for loss-of-function mutations in *mgrB* that reduce its expression or prevent interaction with PhoQ

**Fig 1:**
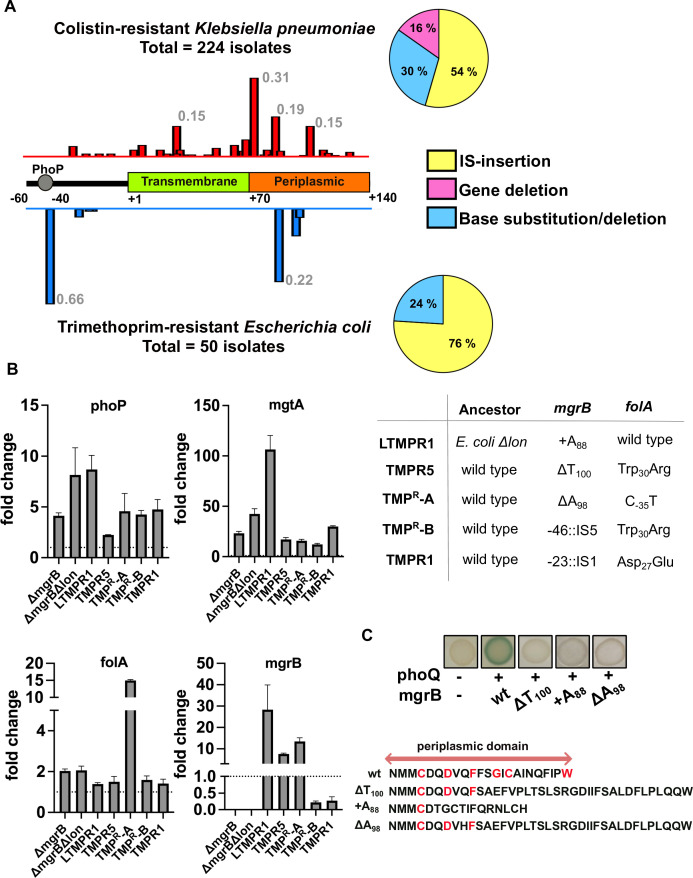
Antibiotic-selected mutations in *mgrB* compromise its function as a feedback regulator of PhoQP signaling. (**A**) Summary of mutations at the *mgrB* locus from 224 colistin-resistant *K. pneumoniae* and 50 trimethoprim-resistant *E. coli* isolates. Percentage of isolates harboring IS-element insertions, single-point mutations, and gene deletions is shown as pie-chart. The location of mutations in *mgrB* or its promoter is shown as a frequency-distribution above (*K. pneumoniae*) and below (*E. coli*) a cartoon of the *mgrB* gene. Peak frequencies corresponding to mutation hotspots are provided. (**B**) Expression levels of *phoP*, *mgtA*, *folA,* and *mgrB* mRNA transcripts measured by quantitative real-time RT-PCR in laboratory-evolved trimethoprim-resistant isolates are expressed as fold-change over appropriate control (wild-type or *E. coli Δlon*). Mutations at *mgrB* and *folA* loci in these isolates are provided for reference. Mutations in non-coding regions and deletions or insertions resulting in a frameshift in *mgrB* are indicated in single-letter nucleotide code (i.e., A, T, G, C). For non-synonymous amino acid substitutions in DHFR, a three-letter amino acid code (i.e., Ala, Trp, etc.) is shown. Fold-change expression of genes in *E. coli ΔmgrB* and *E. coli Δlon ΔmgrB* is shown for comparison. Data represent mean ± S.D. from three independent replicates. No change in expression (=1) is represented by a dotted line. (**C**) Bacterial two-hybrid analysis to test for interaction between mutant MgrBs and wild-type PhoQ. Blue colonies indicate interaction, while white colonies indicate no interaction between MgrB and PhoQ. Representative data from three independent replicates are shown. The sequences of the periplasmic domain of wild-type and mutant MgrB proteins are provided for comparison. Amino acids known to be required for interaction with PhoQ (14) are shown in red.

We compiled data from 17 independent studies describing 224 colistin-resistant *K. pneumoniae* isolates (File S1) (21, 47–63) and 50 laboratory-evolved trimethoprim-resistant *E. coli* isolates from our earlier study (44) to compare the kinds of mutations in *mgrB* from these two systems. Insertion of IS-elements at the *mgrB* locus was the most frequent mutation in both cases (Fig. 1A). In *K. pneumoniae,* IS-insertions disrupting the coding region of *mgrB* were more common, while in *E. coli,* IS-insertions occurred in the promoter between the PhoP-binding box and translation start site (Fig. 1A; File S1). Single-nucleotide deletions/substitutions were the next most frequent and, in both cases, mainly occurred in the region between +70 and +110 bps from the start codon (Fig. 1A; File S1). These mutations either modified specific residues required for PhoQ binding (14) or altered the sequence of the PhoQ-binding periplasmic domain of MgrB by causing a change in the reading frame (File S1). There were also a small number of isolates of *K. pneumoniae* in which the *mgrB* gene was undetectable, indicating a possible deletion (Fig. 1A; File S1).

In both systems, the deletion of *mgrB* is known to confer a fitness advantage upon antibiotic challenge (44, 45, 64). Thus, it was expected that antibiotic-selected *mgrB* mutations in these bacteria are likely to compromise gene function. We tested this prediction for laboratory-evolved trimethoprim-resistant *E. coli* from our strain collection. Since MgrB inhibits PhoQP signaling, we used the expression level of *phoP* and its target *mgtA* as a read-out of MgrB’s function. Indeed, regardless of the type of mutation in *mgrB*, trimethoprim-resistant isolates had higher transcript levels of both genes, comparable to an *mgrB* knockout strain (Fig. 1B). Furthermore, all isolates also had higher expression of *folA*, which we have shown earlier to be associated with trimethoprim tolerance (Fig. 1B). The level of *folA* expression was comparable to an *mgrB* knockout, except when an additional up-regulatory mutation was present in the *folA* promoter (C_-35_T). Next, we measured the expression level of *mgrB* itself in these bacteria. Isolates with an IS-insertion in the promoter of *mgrB* showed an ~80% reduction in transcript levels, explaining their loss-of-function phenotype (Fig. 1B). However, missense mutants of *mgrB* had markedly elevated expression compared to wild type (Fig. 1B). We, therefore, checked whether missense mutants of MgrB retained their ability to interact with PhoQ. Indeed, none of the tested mutants showed any detectable interaction with PhoQ in a bacterial two-hybrid assay (14) (Fig. 1C). Furthermore, replacing the *mgrB* alleles in these isolates with *ΔmgrB::Kan* had no impact on the IC50 of trimethoprim (Fig. S1). Based on these results, we concluded that trimethoprim selected for loss-of-function mutations in *mgrB* and these mutations had two distinct mechanisms, i.e., reducing expression or preventing interaction with PhoQ.

### Mutational loss of *mgrB* facilitates evolution of trimethoprim resistance

By itself, loss of *mgrB* led to a mild enhancement in drug IC_50_ of approximately threefold over wild type (44). Despite this, *mgrB* was consistently mutated across all independently evolved trimethoprim-resistant *E. coli* isolates from our previous work (44). This observation suggested that though *mgrB* deficiency alone had only a small effect on drug resistance, it may serve a facilitatory role during adaptation to trimethoprim. To empirically test this idea, we asked, first, whether it was possible for *E. coli* to evolve resistance without implicating *mgrB,* and second, how *mgrB* mutations impacted the rate of resistance evolution.

**Fig 2:**
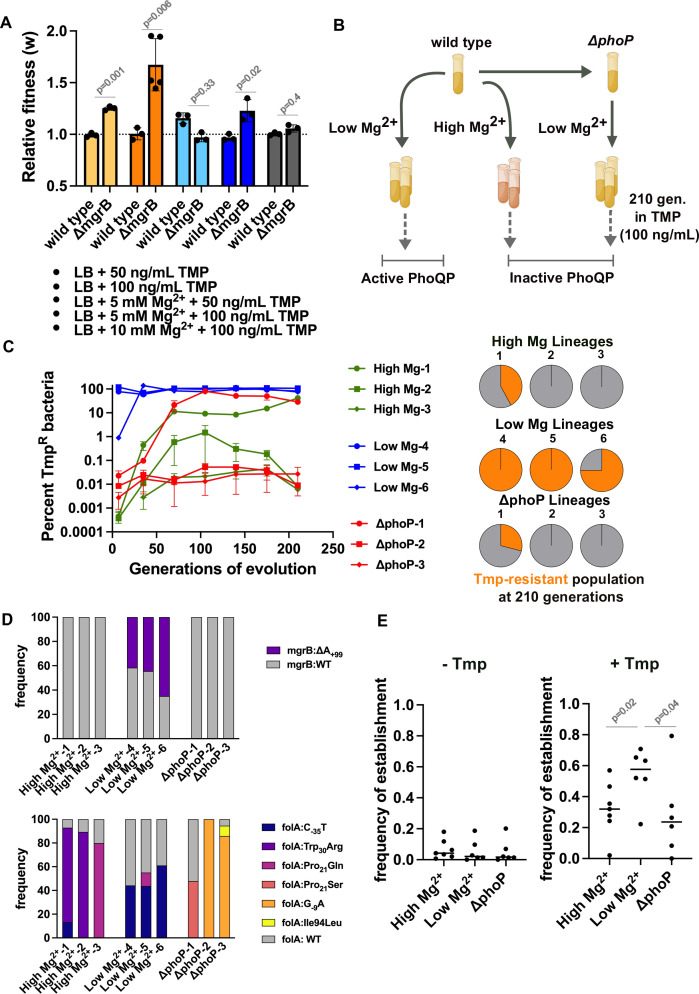
Loss of MgrB facilitates the evolution of trimethoprim resistance in *E. coli*. (**A**) Relative fitness (*w*) of wild-type and *E. coli ΔmgrB*, compared to an *E. coli ΔlacZ* reference strain, in the presence of trimethoprim and MgSO_4_ at the indicated concentrations is plotted. No change in fitness (*w* = 1) is represented by a dotted line. Mean ± S.D. from at least three biological replicates is shown. Statistical significance was tested using an unpaired *t*-test with Welch correction for unequal variances, and *P*-values are shown. (**B**) Schematic for the evolution experiment that investigates the importance of *mgrB* mutations for trimethoprim resistance. Wild-type *E. coli* was evolved in low or high Mg^2+^ media in triplicate for ~210 generations at a trimethoprim pressure of 100 ng/mL (~MIC/9). Similar evolution was also performed for *E. coli ΔphoP* in low Mg^2+^ media. The expected PhoQP activity level in each of the growth conditions is indicated. (**C**) Percentage of trimethoprim-resistant bacteria in 9 evolving lineages over 210 generations. Colony formation at 1 µg/mL (MIC of wild-type *E. coli*) was considered as resistance. Mean ± S.D. of triplicate measurements is plotted. Pie charts show the fraction of the population that had developed resistance at the last time point, i.e., 210 generations, in each of the 9 evolving lineages. (**D**) Frequency of *mgrB* (upper panel) and *folA* (lower panel) alleles at 210 generations of evolution determined by genome sequencing. (**E**) Frequency of establishment of trimethoprim-resistant isolates over a large excess of wild type in drug-free (−Tmp) or 100 ng/mL trimethoprim-supplemented media (+Tmp). A total of 144 competitions were performed for each isolate, and the fraction of replicates in which the resistant isolate outcompeted the wild type is plotted. Median values of six isolates from each evolution condition are shown. Statistical significance was tested using an unpaired *t*-test with Welch correction for unequal variances *t*. The *P*-value is provided.

*E. coli ΔmgrB* showed a dose-dependent increase in relative fitness in trimethoprim-supplemented media (Fig. 2A). High concentration of Mg^2+^ (5–10 mM) in growth media, which is known to inhibit PhoQ activity (11), neutralized this fitness advantage (Fig. 2A). Thus, loss of MgrB was only advantageous for *E. coli* under conditions in which PhoQP was active. Based on this result, we evolved *E. coli* in sub-MIC trimethoprim (100 ng/mL) under conditions of high or low PhoQP activity for ~210 generations (Lineage 1, 2, 3: high Mg^2+^; Lineage 4, 5, 6: low Mg^2+^) (Fig. 2B). We argued that, since PhoQP would be active only at low concentrations of Mg^2+^, these lineages alone would evolve mutations in *mgrB*, allowing us to dissect out its role in the evolution of resistance. In all three low Mg^2+^ lineages, trimethoprim-resistant bacteria were rapidly enriched (i.e., >50% of the population) within the first 50 generations of evolution (Fig. 2C). In contrast, high Mg^2+^ lineages evolved resistance much slower, and resistant bacteria were unable to reach fixation over the duration of the experiment (Fig. 2C). To ensure that this effect was due to the activity of PhoQP and not an independent effect of Mg^2+^, we evolved an isogenic *E. coli ΔphoP* strain in low Mg^2+^ media supplemented with trimethoprim (Fig. 2C). In these populations too, trimethoprim-resistant bacteria were enriched at a slower rate compared to low Mg^2+^ populations and did not get fixed even after 200 generations of evolution (Fig. 2C). Genome sequencing of the evolved populations confirmed that mutations in *mgrB* only evolved under conditions of high PhoQP activity (Fig. 2D; File S2). Importantly, all populations harbored mutations in *folA* at similar frequencies (Fig. 2D; File S2), indicating that the observed differences were not due to different mutation rates at the *folA* locus.

We next calculated the frequency at which two randomly picked resistant isolates from each of the nine evolving lineages could establish over a large excess of a drug-sensitive competitor in the presence and absence of sub-MIC trimethoprim (100 ng/mL) (Fig. 2E). Genome sequencing of these isolates reiterated that *mgrB* mutations were only selected under low Mg^2+^ conditions, while all isolates harbored mutations in *folA* (File S2). Competitions (144 replicates for each strain combination) were set up with wild-type *E. coli* at a starting ratio of 1:10^7^ in favor of wild type to mimic initial stages of evolution when a resistant mutant first emerges in an ancestrally sensitive population. After 24 hours of competition, 0.5% of the mixed culture was passaged into LB media containing trimethoprim at the MIC of wild type (1 µg/mL) (Fig. S2). Growth at this concentration of trimethoprim would indicate that the resistant mutant had been enriched >10-fold during the competition. We noted that all isolates derived from low Mg^2+^ lineages frequently outcompeted the wild type under trimethoprim pressure but not in antibiotic-free media (Fig. 2E). On the other hand, isolates from high Mg^2+^ or *ΔphoP* lineages were more frequently outcompeted by the wild type even in the presence of antibiotic (Fig. 2E). Thus, while *folA*-mutations were sufficient to confer trimethoprim resistance, low/no active PhoQP severely impeded the establishment of trimethoprim-resistant bacteria in *E. coli* populations.

### Pervasive gene deregulation in trimethoprim-resistant bacteria is adaptive in antibiotic-supplemented media

**Fig 3:**
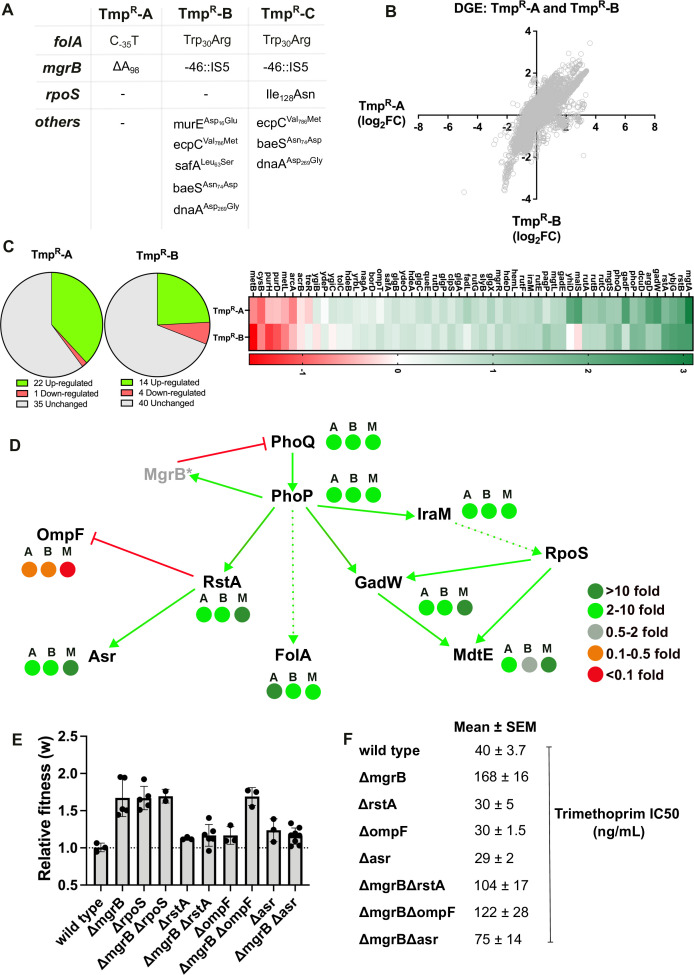
Pervasive deregulation of the gene expression network of *E. coli* in trimethoprim-resistant bacteria is adaptive. (**A**) Mutational profiles of trimethoprim-resistant isolates Tmp^R^-A, B, and C analyzed by RNA-sequencing. (**B**) Correlation between differential gene expression (DGE) profiles of Tmp^R^-A and B. Log_2_(fold change) values of individual genes compared to wild-type *E. coli* in the two resistant isolates are plotted. A linear correlation coefficient of 0.59 revealed highly similar gene expression profiles of the two isolates. (**C**) Expression profile of 58 known targets of PhoP in Tmp^R^-A and B. Pie charts show the fraction of the PhoP-regulated genes that were up-regulated (>twofold), down-regulated (<twofold), or unaltered compared to wild type in the two isolates. The heat map shows log_2_(fold change) based on RNA-seq data in all 58 genes of the PhoP-regulon in Tmp^R^-A and B on a red/green scale. (**D**) PhoP-regulated genes and the downstream network that was altered in Tmp^R^-A and B are shown diagrammatically. The network topology was constructed based on information available on Ecocyc (65). Activation is represented by green arrows, while repression in represented by red lines. Indirect interactions or interactions not involving transcriptional activation are shown as dotted lines. Fold changes in Tmp^R^-A (A), Tmp^R^-B (B), and *ΔmgrB* (M) are shown next to each gene using a red/green color scale. Fold change values for *E. coli ΔmgrB* were taken from Xu et al. (66). (**E**) Contribution of genes downstream of PhoP on the fitness of wild type or *E. coli ΔmgrB* in trimethoprim (100 ng/mL). Relative fitness (*w*) was calculated by direct competition with an *E. coli ΔlacZ* reference strain. No change in fitness (*w* = 1) is shown as a dotted line. Mean ± S.D. of at least three independent replicates is shown. (**F**) Contribution of genes downstream of PhoP on the IC50 of trimethoprim in wild-type or *ΔmgrB* backgrounds. Mean ± S.E.M. of at least three independent replicates is shown.

Genome sequencing from high Mg^2+^ and *ΔphoP* lineages revealed that many isolates harbored mutations in the *folA* promoter ( File S2). These mutations result in several folds higher expression of *folA* than the *mgrB* mutation alone (Fig. 1B). Despite this, their ability to outcompete the wild type was low (Fig. 2E), suggesting that hyperactive PhoQP contributed to higher fitness in trimethoprim by *folA*-independent mechanisms as well. To identify these mechanisms, we analyzed the global gene expression profiles of two independently evolved and isolated trimethoprim-resistant clones from our strain collection by RNA-sequencing (File S3). These isolates, designated Tmp^R^-A and Tmp^R^-B, harbored mutations at the *mgrB* and *folA* loci. Tmp^R^-B also harbored some unique mutations in other genes (Fig. 3A). Differentially expressed genes in both isolates were involved in a wide range of activities, such as carbon and sulfur metabolism, lipopolysaccharide biosynthesis, and transport of metabolites across the cell envelope (Fig. S3). Despite differences in their genotypes, we found that global changes in gene expression were strongly correlated between Tmp^R^-A and Tmp^R^-B (linear correlation coefficient *R*^2^ = 0.59), suggesting that PhoQP hyperactivity may be a major driver for gene deregulation in both isolates (Fig. 3B).

A total of 58 genes are known direct targets of the PhoQP system in *E. coli* (67, 68). We noted that despite the presence of *mgrB*-mutations, a majority of the PhoP-regulon remained unaltered in Tmp^R^-A and B (Fig. 3C and D). For instance, the levels of PhoP targets, such as the *acrAB* efflux pump and the *glg* glycogen metabolism operon, were unaffected by the loss of *mgrB*. These genes are regulated by other transcription factors in addition to PhoP, including master regulators such as CRP (67, 68), and, hence, may be less sensitive to the activation status of PhoP. Hyperactivation of PhoQP was reflected most dramatically in genes that are solely regulated by PhoP, such as the *phoQP* operon itself or the magnesium transporter *mgtA* (Fig. 3C and D). Interestingly, PhoP targets that were up-regulated in Tmp^R^-A and B included other regulators of gene expression such as the *rstAB* two-component system, *gadE* and *gadW* transcription factors, and *iraM* which stabilizes the RpoS sigma factor (Fig. 3C and D). These profiles of expression matched an *mgrB*-deletion strain of *E. coli* reported earlier by Xu et al. (66) (Fig. 3D). We also independently validated these results for selected genes using quantitative RT-PCR in Tmp^R^-A, Tmp^R^-B, *E. coli ΔmgrB,* and *E. coli ΔmgrBΔphoP,* confirming that these changes in gene expression were mediated by hyperactivation of PhoQP (Fig. S4).

Next, we investigated the contribution of two pathways downstream of PhoP that were significantly altered in our RNA-seq experiments toward adaptation to trimethoprim, namely RstA and IraM-RpoS. Deletion of *rpoS* alone enhanced fitness in trimethoprim, while *rstA* deletion alone was neutral (Fig. 3E). In an *mgrB*-knockout background, *rpoS* deletion did not significantly alter fitness upon antibiotic challenge. On the other hand, *rstA*-deficiency reduced, but did not completely neutralize, the fitness advantage of *mgrB* deficiency in trimethoprim (Fig. 3E). *E. coli ΔmgrBΔrstA* also had a lower trimethoprim-IC50 than *E. coli ΔmgrB* (Fig. 3F), although its IC50 values were still higher than wild type. This effect was *folA* independent as *E. coli ΔmgrB* and *E. coli ΔmgrBΔrstA* both overproduced DHFR protein (Fig. S5). RstA is itself a transcription factor downstream of PhoP and is a known repressor of the porin OmpF (69), which enhances entry of antibiotics into the cell. RstA also activates the expression of Asr (69), an acid-responsive periplasmic chaperone associated with response to acid stress (70). We observed that OmpF levels were lower, while Asr was overproduced in Tmp^R^-A and B as expected from higher RstA in these bacteria (Fig. 3D). Other genes known to be regulated by RstA were unaltered in both isolates. Deletion of *ompF* did not detectably alter fitness of *E. coli ΔmgrB* in trimethoprim but led to a mild reduction in drug IC50 (Fig. 3E and F). Interestingly, deletion of *asr* from *E. coli ΔmgrB* reduced fitness to a similar extent as deletion of *rstA* (Fig. 3E). Like *rstA*, though deletion of *asr* reduced the IC50 for trimethoprim, the values continued to be higher than wild type (Fig. 3F). Thus, mutational loss of *mgrB* resulted in hyperactivation of PhoQP, which permeated through the downstream gene regulatory network and extended to secondary and tertiary targets. Specifically, the RstA-target Asr contributed to adaptation, and the net fitness advantage of *mgrB*-loss was derived from at least two independent branches of the PhoP network, namely FolA and RstA-Asr.

### MgrB may be retained by *E. coli* to prevent costs of PhoQP hyperactivation

**Fig 4:**
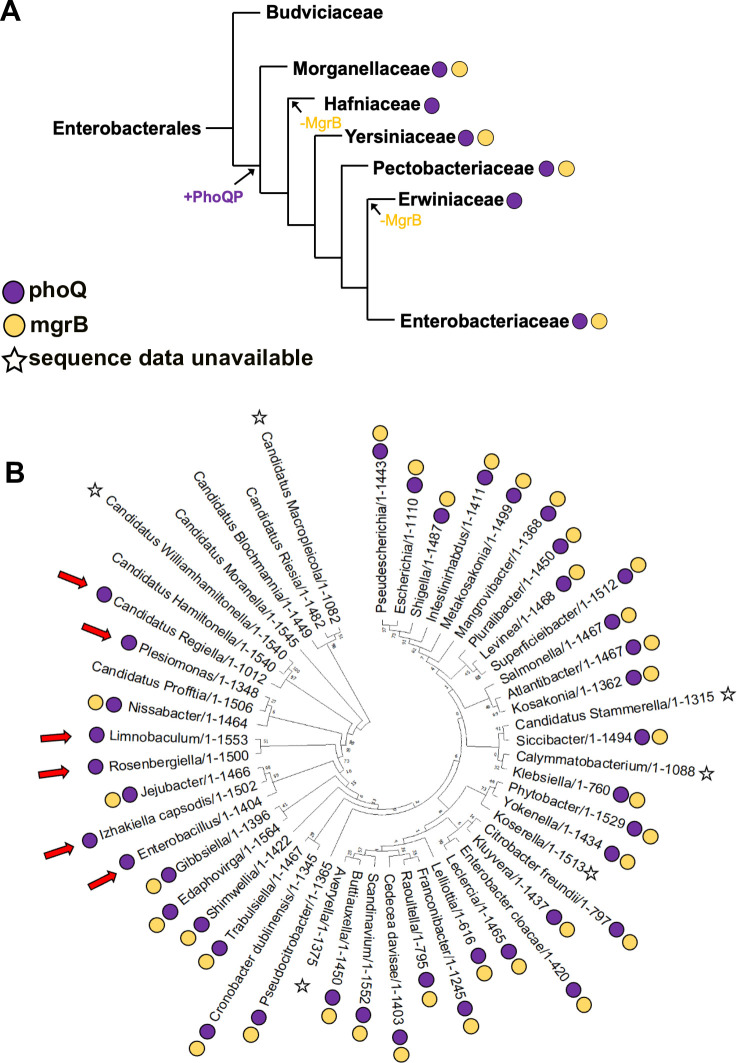
Phylogenetic distribution of *mgrB* and *phoQ* across bacteria. (**A**) Representation of the phylogenetic relationships between seven bacterial families that make up the order Enterobacterales, taken from Adelou et al. (72). The presence of *mgrB* and *phoQ* genes in representative members of these families is shown as yellow and purple circles, respectively. Most probable events of gain of the PhoQP system and loss of MgrB are indicated. (**B**) Maximum likelihood phylogeny of 61 species belonging to family Enterobacteriaceae constructed using 16S rRNA sequences is shown. The presence of *phoQ* and *mgrB* is indicated by purple and yellow circles next to each species. Lack of annotated genome data is indicated by a star. Possible events of *mgrB*-loss are shown as red arrows.

The PhoQP system is restricted to order Enterobacterales. Within this order, the *phoQ* gene is present in all bacterial families except Budviciaceae, indicating that PhoQP may have emerged after its divergence from the other families (Fig. 4A; File S4). Interestingly, the *mgrB* gene is present in four of the six families that harbor *phoQ*, namely Morganellaceae, Yersiniaceae, Pectobacteriaceae, and Enterobacteriaceae but not in Erwiniaceae and Hafniaceae (Fig. 4A; File S4). Phylogenetic relationships between these families suggested that Erwiniaceae and Hafniacieae may have independently lost *mgrB* during evolution. Within the family Enterobacteriaceae, to which *E. coli* belongs, we found that most species harbored both, *phoQ* and *mgrB*. However, a few exceptions, such as Izhakiella, Rosenbergiella, Limnobaculum, and some species of Candidatus, code for PhoQ but not MgrB (Fig. 4B; File S4). Once again, their phylogenetic relationships suggested independent gene-loss events. These analyses showed that sporadic, independent events of loss of the *mgrB* gene have occurred during bacterial evolution. However, most extant bacterial species have preserved a functional *mgrB* gene, indicating that negative feedback in PhoQP is dispensable but desirable. Admittedly, we assume here that the function of MgrB is limited to negative feedback for the PhoQP system and works similarly in different bacterial species. Though there is no evidence to the contrary currently available, the suggestion that there may be species-specific differences in the regulation of PhoP and MgrB has been proposed earlier (71) and remains a caveat of our interpretation of these data.

**Fig 5:**
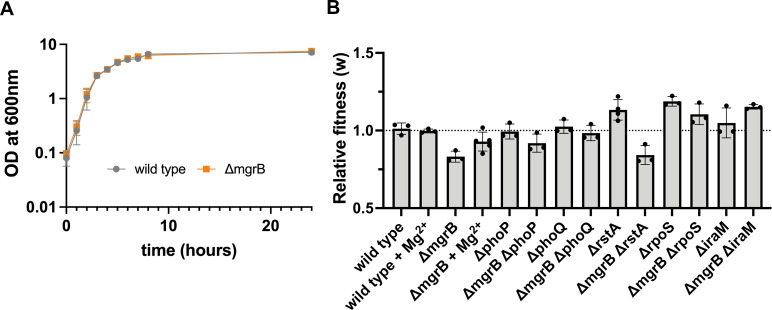
Fitness costs of *mgrB* deficiency in antibiotic-free media. (**A**) Growth of *E. coli* wild type and *ΔmgrB* monitored during growth in drug-free media. Mean ± S.D. of optical density (OD) at 600 nm from triplicate measurements is plotted. (**B**) Relative fitness (*w*) of indicated genotypes in drug-free media compared to an *E. coli ΔlacZ* reference strain is shown. No change in relative fitness (*w* = 1) is shown as a dotted line. Mean ± S.D. from at least three independent replicates is plotted.

The above phylogenetic analyses, together with spontaneous *mgrB*-loss under antibiotic pressure, led us to ask why bacteria like *E. coli* may have retained a functional *mgrB* gene during evolution. There was no detectable difference in the growth characteristics of wild-type *E. coli* and its isogenic *mgrB* knockout strain in antibiotic-free media under standard laboratory conditions (Fig. 5A). However, when *E. coli ΔmgrB* was directly competed against a *ΔlacZ-*marked wild-type strain in drug-free media, a significant fitness cost (*w*_ΔmgrB_ = 0.83 ± 0.03) was observed (Fig. 5B). This fitness cost was due to the hyperactivation of PhoQP since addition of high Mg^2+^ to growth media or deletion of *phoP/Q* restored relative fitness (Fig. 5B). This effect was independent of RstA, as *E. coli ΔmgrBΔrstA* and *E. coli ΔmgrB* had similar relative fitness in drug-free media (Fig. 5B). Our earlier work had shown that loss-of-function mutations in *rpoS* occurred spontaneously during long-term evolution in trimethoprim (44). These mutations improved the fitness of *mgrB*-deficient *E. coli* in drug-supplemented media without affecting the antibiotic’s IC_50_ (44). RpoS is itself an indirect target of PhoQP signaling, and its regulator, IraM, was overproduced in Tmp^R^-A and B as well as in an *mgrB*-knockout strain (Fig. 3C and D). We, therefore, asked whether loss of *rpoS* could compensate for the fitness costs of *mgrB* deficiency in drug-free media as well. Indeed, deletion of *rpoS* or *iraM* restored the relative fitness of an *mgrB*-knockout strain (Fig. 5B). Thus, the costs of *mgrB* deficiency could be mechanistically traced to overproduction of RpoS, possibly explaining why negative feedback may be needed in the PhoQP system.

### RpoS-RpoD imbalance explains the fitness costs of MgrB deficiency in *E. coli*

**Fig 6:**
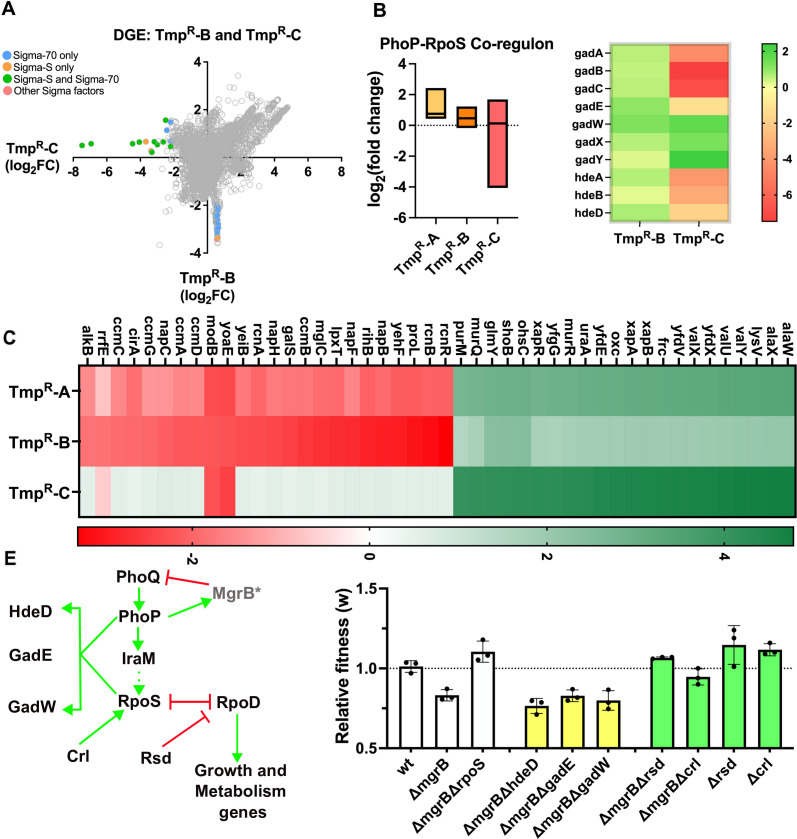
Pervasive deregulation of RpoS-RpoD-regulated genes underlies the fitness costs of *mgrB* deficiency. (**A**) Correlation between differential gene expression (DGE) profiles of Tmp^R^-B and C. Log_2_(fold change) values of individual genes compared to wild-type *E. coli* in the two resistant isolates are plotted. A linear correlation coefficient of 0.19 revealed divergent gene expression profiles of the two isolates. For genes showing greater than fourfold down-regulation in Tmp^R^-B or Tmp^R^-C, data points are colored by the sigma factor responsible for transcription based on data available on Ecocyc (65). (**B**) Expression level of 14 genes co-regulated by PhoP and RpoS in Tmp^R^-A, B, and C strains shown as a box plot (min to max). The median expression level is shown by the line. Genes from the “*gad”* and “*hde”* operons are shown as illustrative of the effects of a mutation in *rpoS* on expression levels of PhoP-RpoS-co-regulated genes. (**C**) Heat map of expression levels of RpoD-target genes in Tmp^R^-A, B, and C strains based on RNA-seq data. For each strain, expression levels were compared to wild type, and log_2_(fold change) is represented on a red/green color scale. (**E**) PhoP-RpoS network and relevant regulators are shown diagrammatically (left panel). Activation is represented by green arrows, while repression is shown by red lines. Dotted line between IraM and RpoS represents indirect effects on protein stability. The topology of the network is drawn based on information available on Ecocyc (65). Relative fitness (*w*) of *E. coli* wild type and indicated gene knockouts compared to an isogenic *lacZ*-knockout reference strain in antibiotic-free media (right panel). Mean ± S.D. values from at least three independent experiments are plotted. No change in relative fitness (*w* = 1) is shown as a dotted line. Yellow bars represent strains that were used to test the contribution of RpoS targets to the costs of *mgrB* deficiency, while green bars represent strains that were used to test the contribution of RpoS-RpoD imbalance to the costs of *mgrB* deficiency.

To investigate the source of fitness costs of *mgrB* deficiency downstream of RpoS, we first analyzed the gene expression profile of a spontaneous trimethoprim-resistant isolate from our strain collection that had a loss-of-function mutation in *rpoS*. This isolate, designated Tmp^R^-C, had a similar mutation profile as Tmp^R^-B and was derived from the same evolving lineage but at a later time point (44), allowing us to assess the specific impact of *rpoS* mutations (Fig. 3A). Despite similarities in their genotypes, Tmp^R^-B and Tmp^R^-C showed significant differences in their transcriptomes (linear correlation coefficient *R*^2^ = 0.19), demonstrating that loss of RpoS substantially rewired the transcriptome of these bacteria (Fig. 6A). We observed that many genes that are co-regulated by PhoP and RpoS were significantly down-regulated in Tmp^R^-C compared to Tmp^R^-B (Fig. 6B and C). This result also held true in *E. coli ΔmgrBΔrpoS*, indicating that loss of *rpoS* overrode the effects of PhoQP hyperactivation for RpoS-PhoP-co-regulated genes (Fig. S4). Additionally, we noted that several differentially expressed genes between Tmp^R^-B and Tmp^R^-C were not direct targets of PhoP or RpoS ( File S2). Particularly striking among them were genes that were significantly down-regulated in both Tmp^R^-A and Tmp^R^-B but restored to wild-type levels in Tmp^R^-C (Fig. 6A and C; File S2). A closer look at this sub-set of genes revealed that a majority of them were transcribed in an RpoD-dependent (i.e., Sigma 70-dependent) manner and were involved in growth and metabolism such as *lpxT* (lipid A metabolism), *ccmA-D* (cytochrome maturation), and *rcnA-B* (metal ion homeostasis) (Fig. 6A and C; File S2). Similarly, several RpoD-regulated tRNA genes were also expressed to a higher level in Tmp^R^-C, compared to Tmp^R^-A and Tmp^R^-B (Fig. 6C; File S2). It is known that RpoS and RpoD compete for the same binding site on RNA polymerase, and competition between these two sigma factors dictates whether *E. coli* expresses genes required for growth and division (RpoD regulated) or stress-response and stationary phase (RpoS regulated) (73, 74). The deregulation of RpoD-target genes in Tmp^R^-A and B thus suggested that precocious RpoS activation due to *mgrB*-deficiency tilted the balance in favor of the RpoS-transcriptional program, which was compensated by mutations in *rpoS*.

Based on the above results, we hypothesized that the cost of *mgrB*-loss may arise due to the overproduction of RpoS-regulated genes and/or the repression of RpoD-regulated genes. To test this prediction, we first generated knockouts of the *hdeD*, *gadW,* and *gadE* genes (co-targets of RpoS and PhoP) in an *mgrB*-deficient background and asked whether these gene deletions could rescue the fitness cost of the *mgrB* knockout (Fig. 6E). These specific genes were selected since they were up-regulated in Tmp^R^-A and B but significantly repressed in Tmp^R^-C (Fig. 6B). However, the fitness of the *mgrB* knockout was unaffected by deletion of these genes (Fig. 6E), ruling them out as the source of the observed fitness cost. To test whether the imbalance between RpoS and RpoD could explain the costs of *mgrB* deficiency, we deleted the *rsd* or *crl* genes from the *mgrB*-knockout and measured relative fitness (Fig. 6E). Rsd is an inhibitor of RpoD (73, 75), while Crl is a potentiator of RpoS (76), and loss of either protein would tilt the balance in favor of RpoD-regulated transcription. Indeed, deletion of *rsd* or *crl* rescued the costs of the *mgrB*-knockout strain (Fig. 6E). These results confirmed that pervasive transcriptional deregulation of gene expression due to hyperactive PhoQP was costly under drug-free conditions and provided a mechanistic explanation for why negative feedback may have been retained by this two-component system during evolution.

## DISCUSSION

Regulatory networks shape the relationship between genotypes and phenotypes by determining which genes are expressed and to what extent. In this study, we have demonstrated that deregulation of the PhoQP pathway by antibiotic-selected mutations in MgrB had a domino-like regulatory impact on several downstream effectors. This pervasive deregulation of gene expression could be adaptive or maladaptive depending on the environmental context. Importantly, the molecular players involved in adaptation and maladaptation were different. In addition to up-regulation of *folA*, the drug target, our experiments identified a new pathway conferring adaptation to trimethoprim, namely activation of the RstA regulon. The RstA-activated acid-inducible chaperone Asr emerged as a major effector of trimethoprim resistance in bacteria with mutant MgrB. On the other hand, the costs of hyperactive PhoQP could be traced to the IraM-RpoS pathway, which in turn led to upsetting the balance of RpoS-RpoD transcriptional programs. Based on the results of the present study, we argue that effectors of antibiotic resistance may be separated from mutation hotspot regulators by several degrees. This separation results directly from the organization of regulatory genes into networks. Mutational rewiring of regulatory networks during evolution modifies their output, ultimately shaping organismal fitness. Understanding the architectures of bacterial gene regulatory networks, and their plasticity, is therefore crucial to deciphering how regulatory mutations confer AMR in bacterial pathogens.

A key finding from the present study is the role of the RstA-Asr pathway in enhancing fitness in trimethoprim. This pathway has been associated with tolerance to acid stress and is induced by mildly acidic conditions (69, 77, 78). Asr’s chaperone activities in the periplasm are required for its protective function against acid due to its role in cell envelope remodeling and protein export (70), although the precise molecular mechanisms remain to be elucidated. The induction of Asr is, however, not specific to acid and occurs in a number of stresses such as heavy metal toxicity and oxidative damage (70). Interestingly, induction of the RstA-Asr pathway by mild acid is mediated by PhoQP activation (69, 78), and our results show that *E. coli* under trimethoprim stress evolves to co-opt this pathway for adaptation to antibiotic. Though we have not investigated how up-regulation of Asr contributed to trimethoprim adaptation, we propose that it may be due to a general enhancement in stress tolerance.

A few earlier studies have demonstrated parallels between responses to acid and trimethoprim in *E. coli*. Mitosch et al. found that trimethoprim treatment elicited an acid-stress response in *E. coli* and that a drop in intracellular pH due to nucleotide depletion was correlated with poor survival in the antibiotic (79). Similarly, *asr* was one of the hits in a screen for mutants of *E. coli* that fared poorly in a model of cell death due to thymidine starvation (80). Taken together with our results, we propose that trimethoprim-induced thymidine starvation mimics mild acid stress, which may be mitigated by proteins like Asr that combat cytosolic acidification. We note that *E. coli* has at least four different acid resistance (AR) pathways, namely RpoS and glucose-dependent AR1, glutamate-dependent AR2, arginine-dependent AR3, and lysine-dependent AR4 (81). Based on RNA-sequencing of Tmp^R^-A, B, and C, it is clear that components of AR3 and AR4 pathways are unaltered in trimethoprim-resistant isolates ( File 3). We also note that AR1 and AR2 pathways in *E. coli* rely on RpoS-dependent gene expression (80, 81). However, our genetic analyses show that this branch may not be relevant for survival in trimethoprim. Likewise, the RstA-Asr pathway was only beneficial in trimethoprim when it was hyperactivated in *mgrB*-mutant bacteria and not under basal levels of activity. Thus, while there is an overlap between responses to trimethoprim and acid, there are only specific components of the latter that are beneficial in the antibiotic. Importantly, the present study sheds light on the ability of bacteria to repurpose existing stress response pathways to combat antibiotic pressure. Greater insight into the relationship between stress response and antibiotic evasion may identify new points of intervention for therapeutic design in the future.

Beyond antibiotic resistance, our study answers why negative feedback may emerge in gene regulatory networks from an evolutionary perspective. The only empirically validated basis for the evolution of negative feedback in transcriptional networks is to enhance mutational tolerance. Marciano et al. used the LexA transcription factor of *E. coli* to demonstrate that negative feedback canalizes phenotypes and provides greater tolerance to mutational perturbation (82). Similar observations were made for the Rox1 protein from *Saccharomyces cerevisiae*, where negative feedback stabilized gene expression levels and enhanced mutational robustness (83). For the PhoQP system, it has been speculated that MgrB may be needed to optimize signaling output in environments with varying Mg^2+^ concentrations (40–42). Though our evolution and fitness experiments do not account for the effects of dynamically fluctuating Mg^2+^ concentrations on bacterial fitness, we provide an alternative explanation for why negative feedback may have been maintained by *E. coli* in PhoQP, i.e., to prevent pervasive deregulation of the gene regulatory network. We note that extrapolating this idea from *E. coli* to other bacteria is contingent on whether perturbing PhoQP has widespread gene regulatory impact in different bacterial species. Some studies have demonstrated this to be true. For example, in *Pectobacterium versatile* and *Yersinia pestis*, genome-wide transcriptomics and ChIP-seq have revealed that PhoP influences the expression of several genes beyond its direct targets, although the mechanisms are not yet clear (84–86). Thus, we propose that negative feedback may serve to insulate regulatory pathways that can potentially cross-activate, like PhoQP and RpoS transcriptional networks.

It is important to note here that the requirement for negative feedback is highly contextual in pathways like PhoQP that directly respond to the extracellular medium. Indeed, the deregulation of PhoQP by loss of MgrB is itself beneficial in many environments such as acid stress, antimicrobial peptides, and antibiotics (44, 48, 66, 85, 87). Our results demonstrate that the benefits of PhoQP deregulation are derived from a network-level response involving multiple molecular players. Similarly, cross-activation of other regulatory pathways by PhoQP, though costly under the conditions tested by us, may have advantages in more complex environments. Bray et al. demonstrated that the loss of MgrB in colistin-resistant *K. pneumoniae* is accompanied by compromised gastrointestinal colonization efficacy (64). Like our results, this study too reported the overproduction of RpoS in *mgrB*-deficient *K. pneumoniae*. However, the authors found that RpoS overproduction was beneficial in a model for pathogen transmission (64). Furthermore, it could be envisaged that activation of PhoQP by a decrease in intracellular or extracellular pH (88) may collaterally confer benefits in trimethoprim, and vice versa. Similarly, activation of the PhoQP pathway by antimicrobial peptides (87) may collaterally enhance trimethoprim and acid tolerance. These possibilities have a bearing on antibiotic susceptibilities of “naïve” enterobacterial populations that have not been exposed to antibiotics, such as those facing acidic pH in a human host or in soil and water. Thus, life history and growth context are both likely to have a strong influence on how deregulation of gene expression translates to organismal fitness.

**Fig 7:**
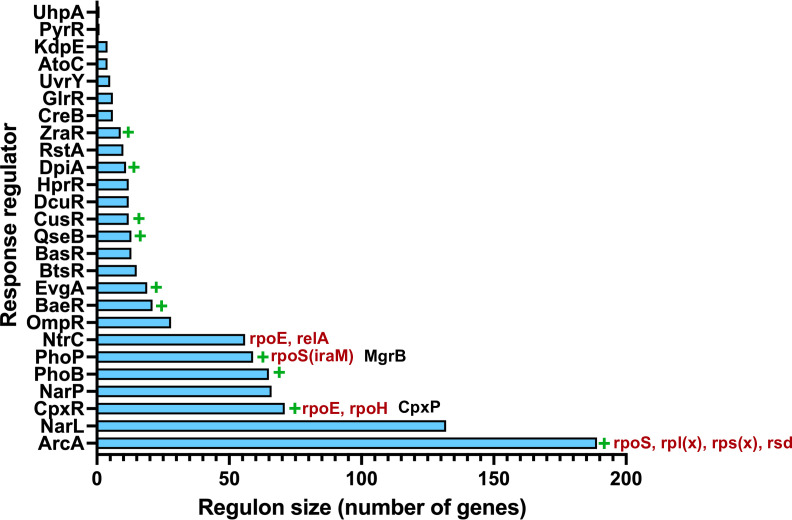
Regulon size and feedback in two-component systems of *E. coli*. Response regulators of 26 two-component systems from *E. coli* K-12 MG1655 ranked by their regulon sizes. Green plus sign (+) indicates that the two-component system is known to have positive feedback, i.e., stimulate its own transcription. Global regulators cross-activated by these systems are in crimson next to respective response regulators, while known negative feedback regulatory proteins are in black. All information collated from Ecocyc (65).

Curiously, the evolution of negative feedback proteins in two-component signaling pathways appears to be the exception rather than the rule. In *E. coli,* only two systems, i.e., PhoQP and CpxAR, have known negative feedback regulator proteins (89). Based on the idea that pervasive gene deregulation drives the evolution of negative feedback, we propose that the following criteria necessitate the evolution of negative feedback in two-component pathways. First, the system must have a large regulon which increases the chance of its activity translating to changes in organismal fitness across environments. Alternatively, the system’s regulon must include genes that can produce large fitness effects upon overexpression. This is exemplified by the CpxAR pathway, which when hyperactivated results in severe growth and division defects due to the overproduction of LdtD, a peptidoglycan cross-linking enzyme (90). Likewise, ectopic production of RcsB, the response regulator of the Rcs system, leads to a disbalance of cell growth and division by affecting nucleoid compaction and FtsZ positioning (91). Second, the system should have a positive feedback loop, i.e., it should activate its own expression. This is known to be true for the PhoQP system and results in rapid amplification of signaling after activation (41). Finally, the regulon of the two-component system should be connected to other global regulatory networks, such as RpoS in the case of PhoQP. For *E. coli*, only three two-component systems satisfy all these criteria, namely PhoQP, CpxAR, and ArcAB, of which two have known negative feedback regulators (Fig. 7). In the case of ArcAB, we are not aware of negative feedback systems; however, we believe that it may be reasonable to look for them in the future. We cannot rule out the role of regulatory RNAs here, several of which are known to be activated by two-component signaling (92). Further investigation would be needed to test whether their contribution is similar to that of MgrB.

In conclusion, despite extensive mechanistic insights into the functioning of gene regulatory networks, there is relatively little known about how they evolve and are rewired in response to environmental perturbation. Using the PhoQP-MgrB system of *E. coli* evolving in trimethoprim, we have analyzed the proximal and distal effects of loss of negative feedback on the cellular transcriptional network. By linking these changes with organismal fitness across relevant environments, we have shown how evolution in a gene regulatory network proceeds in response to selection and can drive and modulate bacterial adaptation.

## MATERIALS AND METHODS

### Bacterial strains and culture conditions

*E. coli* wild type and its mutants were cultured in Luria-Bertani broth (LB) or on Luria-Bertani agar (LA plates). Media were supplemented with trimethoprim and MgSO_4_ at required concentrations as needed. Kanamycin or chloramphenicol for selection of transformants/knockouts was added to growth media at 30 µg/mL each as needed. Ampicillin was used at 100 µg/mL.

The strains and plasmids used in this study are shown in Table 1.

**TABLE 1:** List of strains and plasmids used in this study

Sr. no.	Strain	Description	Source
1.	*Escherichia coli* K-12 MG1655	Wild-type *E. coli*; used as ancestor for evolution experiments and as reference strains for phenotypic and genotypic comparisons	Kind gift from Prof. Sutirth Dey, IISER (Pune)
2.	*E. coli SAM85*	*cya^−^* host strain for bacterial two-hybrid assay	Kind gift from Prof. Mark Goulian, University of Pennsylvania (USA) (14, 40)
Gene knockouts generated in E. coli K-12 MG1655			
3.	*E. coli ΔmgrB*	Isogenic knockout of *mgrB* gene (*ΔmgrB::Kan*)	(44)
4.	*E. coli ΔlacZ*	Isogenic knockout of *lacZ* gene (*ΔlacZ::Cat*)	(44)
5.	*E. coli ΔphoP*	Isogenic knockout of *phoP* gene (*ΔphoP::Kan*)	(44)
6.	*E. coli ΔphoQ*	Isogenic knockout of *phoQ* gene (*ΔphoQ::Kan*)	(44)
7.	*E. coli ΔiraM*	Isogenic knockout of *iraM* gene (*ΔiraM::Kan*)	(44)
8.	*E. coli ΔrpoS*	Isogenic knockout of *rpoS* gene (*ΔrpoS::Kan*)	(44)
9.	*E. coli Δrsd*	Isogenic knockout of *rsd* gene (*Δrsd::Kan*)	This study
10.	*E. coli Δcrl*	Isogenic knockout of *crl* gene (*Δcrl::Kan*)	This study
11.	*E. coli ΔrstA*	Isogenic knockout of *rstA* gene (*ΔrstA::Kan*)	This study
12.	*E. coli ΔompF*	Isogenic knockout of *ompF* gene (*ΔompF::Kan*)	This study
13.	*E. coli Δasr*	Isogenic knockout of *asr* gene (*Δasr::Kan*)	This study
14.	*E. coli ΔmgrBΔphoP*	Double knockout of *mgrB (ΔmgrB::FRT*) and *phoP (ΔphoP::Kan*) genes	(44)
15.	*E. coli ΔmgrBΔphoQ*	Double knockout of *mgrB (ΔmgrB::FRT*) and *phoQ (ΔphoQ::Kan*) genes	(44)
16.	*E. coli ΔmgrBΔiraM*	Double knockout of *mgrB (ΔmgrB::FRT*) and *iraM (ΔiraM::Kan*) genes	This study
17.	*E. coli ΔmgrBΔrpoS*	Double knockout of *mgrB (ΔmgrB::FRT*) and *rpoS (ΔrpoS::Kan*) genes	This study
18.	*E. coli ΔmgrBΔrsd*	Double knockout of *mgrB (ΔmgrB::FRT*) and *rsd (Δrsd::Kan*) genes	This study
19.	*E. coli ΔmgrBΔcrl*	Double knockout of *mgrB (ΔmgrB::FRT*) and *crl (Δcrl::Kan*) genes	This study
20.	*E. coli ΔmgrBΔhdeD*	Double knockout of *mgrB (ΔmgrB::FRT*) and *hdeD (ΔhdeD::Kan*) genes	This study
21.	*E. coli ΔmgrBΔgadE*	Double knockout of *mgrB (ΔmgrB::FRT*) and *gadE (ΔgadE::Kan*) genes	This study
22.	*E. coli ΔmgrBΔgadW*	Double knockout of *mgrB (ΔmgrB::FRT*) and *gadW (ΔgadW::Kan*) genes	This study
23.	*E. coli ΔmgrBΔrstA*	Double knockout of *mgrB (ΔmgrB::FRT*) and *rstA (ΔrstA::Kan*) genes	This study
24.	*E. coli ΔmgrBΔompF*	Double knockout of *mgrB (ΔmgrB::FRT*) and *ompF (ΔompF::Kan*) genes	This study
25.	*E. coli ΔmgrBΔasr*	Double knockout of *mgrB (ΔmgrB::FRT*) and *asr (Δasr::Kan*) genes	This study
Strains/isolates derived from adaptive laboratory evolution used for gene expression analysis			
26.	Tmp^R^-A	Trimethoprim-resistant isolate derived from long-term evolution harboring *folA-C_-35_T* and *mgrB-ΔA_98_* mutations	(44)
27.	Tmp^R^-B	Trimethoprim-resistant isolate derived from long-term evolution harboring *folA-Trp_30_Arg* and *mgrB::IS5_-46_* mutations	(44)
28.	Tmp^R^-C	Trimethoprim-resistant isolate derived from long-term evolution harboring *folA-Trp_30_Arg*, *mgrB::IS5_-46_,* and *rpoS-Ile_128_Asn* mutations	(44)
29.	LTMPR1	Trimethoprim-resistant isolate derived from long-term evolution harboring wild-type *folA* and *mgrB + A_98_* mutation in a *Δlon::Kan* background	(44)
30.	TMPR1	Trimethoprim-resistant isolate derived from short-term evolution harboring *folA-*D27E and *mgrB*::IS1_-23_ mutations	(44)
31.	TMPR5	Trimethoprim-resistant isolate derived from short-term evolution harboring *folA-*W30R and *mgrB ΔT_100_* mutations	(44)
Plasmids for bacterial two-hybrid assay			
Sr. no.	Plasmid	Description	Source
1.	pAL27	pKT25 P_lac_-*cyaA_T25_-phoQ*, Kan^R^	Kind gift from Prof. Mark Goulian, University of Pennsylvania (USA) (14, 40)
2.	pAL33	pUT18C P_lac_-*cyaA_T18_-mgrB*, Amp^R^	Kind gift from Prof. Mark Goulian, University of Pennsylvania (USA) (14, 40)
3.	pUT18C	pUC19 P_lac_-*cyaA_T18_*-MCS, Amp^R^	Kind gift from Prof. Mark Goulian, University of Pennsylvania (USA) (14, 40)
4.	pUT18C-*mgrB-ΔT_100_*	pUC19 P_lac_-*cyaA_T18_- mgrB-ΔT_100_*, Amp^R^	This study
5.	pUT18C-*mgrB-+A_88_*	pUC19 P_lac_-*cyaA_T18_- mgrB-+A_88_*, Amp^R^	This study
6.	pUT18C-*mgrB-ΔA_98_*	pUC19 P_lac_-*cyaA_T18_- mgrB-ΔA_98_*, Amp^R^	This study
7.	pBAD33-*mgrB-ΔT_100_*	pBAD33 with *mgrB-ΔT_100_* Chloramphenicol^R^	This study
8.	pBAD33- *mgrB-+A_88_*	pBAD33 with *mgrB-+A_88_* Chloramphenicol^R^	This study
9.	pBAD33- *mgrB-ΔA_98_*	pBAD33 with *mgrB-ΔA_98_* Chloramphenicol^R^	This study

### Relative fitness measurements

Relative fitness (*w*) of various mutant strains of *E. coli* was measured by direct competition with an *E. coli ΔlacZ* strain under appropriate growth conditions. Neutrality of the *ΔlacZ* genetic marker was established by competitions with unmarked wild-type *E. coli* under every growth condition tested. The detailed methodology followed is described in Patel and Matange (44). Briefly, test and reference strains were grown overnight in LB till saturation. Cultures were mixed 1:1, or with an appropriate bias as needed, in 3 mL LB supplemented with appropriate antibiotic concentrations. Initial CFU/mL of test and reference strains was determined by serially diluting the mixed culture and plating on LA supplemented with IPTG (1 mM) and X-Gal (50 µg/mL). The strains were allowed to compete for 20–24 hours (approximately seven generations), and CFU/mL was determined once again. Relative fitness (*w*) was calculated using the following formula:


$$w=\ln\left(\frac{Tf}{Ti}\right)/ln\left(\frac{Rf}{Ri}\right)$$

where *Tf* and *Ti* are the final and initial CFU/mL of the test strain, and *Rf* and *Ri* are the final and initial CFU/mL of the reference strain, respectively.

### Monoculture growth curves

Bacterial strains to be characterized were initially grown to saturation overnight. From saturated cultures, bacteria were passaged (0.1%) into 5 mL LB broth and grown at 37°C, with shaking at 180 rpm. Aliquots were taken for measuring bacterial growth periodically until 20–24 hours of growth. Bacterial growth was monitored using optical density (OD) at 600 nm.

### Bacterial two-hybrid assay

To test the interaction between PhoQ and MgrB mutants, a bacterial two-hybrid assay developed for this system (14) was adapted. Mutant *mgrB* genes were cloned downstream of, and in frame with *Bordetella pertussis cyaT18* fragment, in pUT18C. These clones were co-transformed with pAL27, which contains wild-type *phoQ* downstream of and in-frame with *cyaT25* fragment, in *E. coli* SAM85. Strains with wild-type *phoQ* and *mgrB*, along with single transformants, were used as controls. To perform the assay, the transformed strains were inoculated in 1 mL LB and allowed to grow at 37°C for 6 hours. Post incubation, 10 µL of the undiluted culture was spotted on LB agar plates containing IPTG (1 mM) and X-Gal (50 µg/mL). The spots were dried, and plates were incubated at 37°C for ~17 hours. Color of the spots was noted post incubation. Blue-colored colonies indicated interaction between the two proteins, whereas white-colored colonies indicated no interaction.

#### Cloning of mutant *mgrB* alleles into pUT18C

Mutant *mgrB* alleles were first amplified by PCR using genomic DNA from trimethoprim-resistant isolates as template and mgrB_pBAD33_fwd (5′-ATGCGTCGAC GTGAAAAAGTTTCGATGGGT-3′) and mgrB_pBAD33_rev (5′- ATGCAAGCTT AGTAGAGGCGCTATTCTACC-3′) primers. Amplified DNA was digested with *Sal*I and *Hind*III restriction endonucleases and ligated with similarly digested pBAD33 plasmid. The sequences of positive clones were confirmed by sequencing and restriction digestion. These clones were then used as the template to amplify *mgrB* mutants using mgrB_BACTH_XbaI_fwd (5′-ACATCTAGAGATGAAAAAGTTTCGATGGG-3′) and mgrB_BACTH_KpnI_rev (5′-ATGCGGTACCAGTAGAGGCGCTATTCTACC-3′) primers. Amplified DNA was digested with *Xba*I and *Kpn*I, and ligated with similarly digested pUT18C, to give the following clones: pUT18C-*mgrB-ΔT_100_*, pUT18C-*mgrB-+A_88_,* and pUT18C-*mgrB-ΔA_98_*.

### Immunoblotting

DHFR protein levels were estimated by immunoblotting using anti-DHFR polyclonal IgG as described in Patel and Matange (44). Briefly, equal total cellular protein (5 µg) was electrophoresed using SDS-PAGE on a 15% gel. Proteins were then electroblotted onto a PVDF membrane. The membrane was blocked using 5% BSA solution and cut into two halves at the 25-kDa-molecular-weight band. The lower part of the membrane was probed with anti-DHFR polyclonal IgG (100 ng/mL), and the upper part was probed with anti-FtsZ serum (1:50,000). HRP-linked anti-rabbit IgG (1:10,000) was used as the secondary antibody, and hybridized antibodies were detected using chemiluminescence.

### Laboratory evolution of trimethoprim resistance

The detailed methodology for laboratory evolution of trimethoprim resistance is described in Patel and Matange (44) and Vinchhi et al. (93). Briefly, triplicate bacterial populations were grown in trimethoprim supplemented LB (low Mg^2+^) or LB + 10 mM MgSO_4_ as required for 15–18 hours before passaging (1%) into fresh media. Bacteria were passaged for ~210 generations (6–7 generations per growth cycle), and aliquots were periodically frozen at −80°C for further analyses. Trimethoprim was used at a concentration of 100 ng/mL throughout the experiment, which corresponds to ~MIC/9.

The titers of trimethoprim-resistant bacteria at different generations of evolution were determined by reviving frozen stocks in drug-free LB overnight until saturation, preparing a 10-fold serial dilution of each culture and then spotting 10 µL of each dilution on LA supplemented with 1 µg/mL of trimethoprim (MIC of wild type). Plates were incubated for ~20 hours before counting colonies and estimating CFU/mL.

### Genome sequencing of laboratory-evolved trimethoprim-resistant isolates

Genome sequencing and variant calling for evolved bacteria were carried out as described in Patel and Matange (44) and Vinchhi et al. (93). Briefly, trimethoprim-resistant clones or evolving populations were revived from frozen stocks into 3 mL of LB and grown overnight until saturation. Genomic DNA was extracted from bacterial pellets using phenol:chloroform:isoamyl alcohol and cleaned up using spin-columns (Takara, Japan). Paired-end whole-genome next-generation sequencing was performed on a MiSeq system (Illumina, USA) with read lengths of 150 bps. Library preparation and sequencing services were provided by Eurofins, India. Processed reads were aligned to the reference genome *E. coli* K-12 MG1655 (U00096) using bowtie2. Variant calling and prediction of new junctions in the genome to identify large structural mutations were done using Breseq, using default settings in population or clonal modes as needed (94). All variants and new junctions that were already present in the wild-type ancestor were excluded.

### Genetic manipulations and gene knockout in *E. coli*

All gene knockouts were generated using P1 transduction by moving kanamycin-resistance-marked gene deletions from donor strains taken from the Keio Collection (95, 96) into appropriate recipient strains. Knockouts were confirmed using gene-specific PCR on genomic DNA extracted from transductants. Double knockouts were generated by first removing the kanamycin resistance cassette from *E. coli* single-knockout strains using the pCP20 plasmid expressing the FLP recombinase (97) and then introducing the second knockout allele by P1 transduction. For unmarking knockouts, the pCP20 plasmid was transformed into chemically competent knockout strains. Transformants were selected on LA plates containing ampicillin at 30°C. Colonies from ampicillin-supplemented plates were streaked on LA, LA-kanamycin, and LA-ampicillin to ensure loss of kanamycin resistance and pCP20. Unmarked strains were confirmed by colony PCR using gene-specific primers.

### Measuring trimethoprim resistance

Trimethoprim resistance of *E. coli* mutants and resistant isolates was measured using a broth dilution assay. Appropriate strains of *E. coli* were grown for 6–8 hours at 37°C and then inoculated (1:100) into serially diluted trimethoprim-containing media in a total volume of 150 µL per well. Optical density at 600 nm was measured after 18–20 hours of incubation at 37°C. All data were normalized to OD values in the absence of antibiotic (set to 1), and experimental data were fit to a log(inhibitor)-response model using Graphpad Prism (version 9.1.4). This model fits data to a sigmoidal curve and is described by the following equation:


$$Y=\;Min\;+\;\left(Max-Min\right)/\left(1+10^{\left(X-Log\left(IC50\right)\right)}\right)$$

where

Min: minimum value of normalized OD and Max: maximum value of normalized OD.

From this model fitting, values of IC50, i.e., drug concentration needed to achieve 50% growth inhibition, were estimated.

### Establishment propensity of trimethoprim-resistant isolates at sub-MIC drug pressures

To estimate the propensity of establishment of trimethoprim-resistant isolates, their ability to outcompete a large excess of wild-type *E. coli* in sub-MIC trimethoprim was evaluated (Fig. S2). To set up the primary competitions, trimethoprim-evolved isolates and wild-type *E. coli* were mixed (1:10^7^), and the mixture was diluted 100 times in a final volume of 15 mL LB broth supplemented with 100 ng/mL trimethoprim. This diluted culture was then dispensed into the wells of a sterile 96-well polystyrene plates (200 µL per well) and incubated at 37°C for 24 hours, with shaking at 180 rpm. Next, 1 µL from each well was passaged into 200 µL of LB broth containing 1 µg/mL trimethoprim (i.e., MIC of wild type) in a fresh 96-well plate. This plate was incubated at 37°C for 24 hours with shaking at 180 rpm. Only those wells in which mutant bacteria were enriched by >10-fold would show turbidity. The number of wells showing visible growth was noted, and OD_600_ was measured using a plate reader. Two controls were also set up in parallel. For the first control, primary competition was performed in the absence of trimethoprim such that there would be no enrichment of resistant mutants. The second control had only wild-type bacteria. This control would indicate the frequency of spontaneous resistant mutants emerging during the competition. A value of OD_600_ greater than 0.5 in trimethoprim was used as the threshold for establishment of the mutant over wild type.

### Bioinformatics and phylogenetic analyses

To analyze the distribution of homologs of PhoQ and MgrB from *E. coli* across different bacterial species, the Pfam database (98) was initially mined. Since both proteins were restricted to the order Enterobacterales, further analyses were restricted to this group of bacteria. The phylogenetic relationship of different families under the order Enterobacterales was taken from Adelou et al. (72). Type strains from each family were identified using the LPSN (List of Prokaryotic names with Standing in Nomenclature; https://lpsn.dsmz.de/) database (99, 100). For family Enterobacteriaceae, a maximum likelihood phylogenetic tree was constructed using 61 bacterial species in MEGA-X (101), by using 16S rRNA gene sequences of the type strain of each genus as listed in LPSN (99, 100). The presence or absence of PhoQ and MgrB in bacterial strains under consideration was determined by three methods: first, directly analyzing genome annotation data available on NCBI, second, by performing BLASTn against the genome of the query organism (nucleotide similarity with wild-type *E. coli mgrB*-NCBI Gene ID: 946351 and *phoQ*-NCBI Gene ID: 946326), third, by performing NCBI BLASTp against the proteome of the query organism (amino acid similarity with wild-type *E. coli* MgrB-Uniprot: P64512 and PhoQ-Uniprot: P23837). A positive hit in at least one of the above searches was taken to mean that PhoQ/MgrB was present in the query organism.

### Gene expression analysis

#### Transcriptome analyses using RNA-sequencing

For RNA-seq, 1% of a saturated culture of wild-type and trimethoprim-resistant isolates Tmp^R^-A, Tmp^R^-B, and Tmp^R^-C was inoculated into 3 mL LB in duplicate and incubated at 37°C for 3 hours with shaking at 180 rpm. Bacteria were pelleted down by centrifugation and resuspended in 1 mL of RNAlater (Invitrogen) for storage. Subsequent RNA extraction, sequencing, and preliminary data analyses were performed by Redcliff Life Sciences (India). Total RNA was extracted using RNAeasy spin columns and quantitated using Qubit and Bioanalyzer. Bacterial rRNA was depleted using Ribozero Kit. Bacterial mRNA was then reverse transcribed; library was prepared, and quality control was performed using Tapestation platform. Paired-end sequencing was performed on Illumina platform with read length of 150 bp. Processed reads were aligned to the reference genome (U00096) using HISTAT2 (version 2.1.0). Abundance estimation was done using featureCounts (version1.34.0). Differential gene expression (DGE) analysis was done by comparing the expression of individual gene trimethoprim-resistant strains to wild type using DESeq2. The output of the DGE analyses was log_2_(fold change) values for each gene and *P*-values to test for statistical significance. Functional annotation of pathway up- or down-regulated was done using DAVID (102). Differentially expressed genes were classified into PhoP regulated, RpoS regulated, and RpoD regulated based on information available in RegulonDB (67, 68) and Ecocyc (65). Sigma factors regulating differentially expressed genes were also obtained from RegulonDB (67, 68) and Ecocyc (65).

#### Gene-specific quantitative RT-PCR

Quantitative RT-PCR was used to validate the findings of RNA-seq. For RNA extraction, appropriate bacterial strains were grown for 3 hours at 37°C and pelleted by centrifugation. Total RNA was extracted using TRIzol Reagent (Invitrogen, USA) and quantified spectrophotometrically. RNA quality was evaluated by electrophoresis on a 1% agarose gel and staining with ethidium bromide. Extracted RNA (20 µg) was treated with recombinant DNase I (RNAse free) (Takara, Japan) and then cleaned up using RNeasy spin column (Qiagen, Japan). Prepared RNA was stored at −80°C until further use. Reverse transcription reaction was set up using the cleaned-up RNA (~1.6 µg) using PrimeScript RT Reagent Kit (Takara, Japan). The RT reaction was carried out with buffering temperature at 25°C for 2 min and cDNA synthesis at 37°C for 30 min. The RT enzyme was heat inactivated at 85°C for 90 seconds. Prepared cDNA and RT controls were serially diluted (10-, 100-, and 1,000-fold), and semi-quantitative PCRs were set up using gene-specific primers (Table 2) to ensure the absence of contaminating genomic DNA and checking for priming efficiency. cDNA was stored at −20°C until further use.

**TABLE 2:** List of primer, cDNA dilution, and expected product sizes for qPCR

Sr. no.	Gene	Primer sequence (5′–3′)	Amplicon length (bp)	cDNA dilution used for qPCR
1.	16SrRNA(*rrnA*)	Fwd: GCTACAATGGCGCATACAAA	101	1:1,000
		Rev: TTCATGGAGTCGAGTTGCAG		
2.	*mgtA*	Fwd: CGGCACAAGCAATGGTGATT	151	1:10
		Rev: GCCATCACCAGCATAAAGCG		
3.	*mgrB*	Fwd: AGTTTCGATGGGTCGTTCTGG	122	undiluted
		Rev: CTGGTTAATGGCACAAATTCCG		
4.	*hdeD*	Fwd: GTGTTGCAGGGGTTATTCGC	142	1:10
		Rev: AGCGTTACAGACACCATCGG		
5.	*gadW*	Fwd: GCTCGGTGATCCTCATTCGT	147	1:10
		Rev: CGATATCCAGTCGTCGCGTA		
6.	*phoP*	Fwd: TCCGAGATCGACAATCGCAA	164	1:10
		Rev: GCGCGTACTGGTTGTTGAAG		
7.	*acrA*	Fwd: TACGCGCTATCTTCCCGAAC	154	Undiluted
		Rev: CAACTACCAGTACGGTGGCA		
8.	*folA*	Fwd: GTAACGTGGGTGAAGTCGGT	143	Undiluted
		Rev: ACTTCTGCGTCGATATGCGT		

Quantitative RT-PCR (qPCR) for target genes was performed using TB GreenPremix Ex Taq II (Tli RNaseH Plus) (Takara, Japan) and using gene-specific primers listed in Table 2. For each PCR, 20 µL reaction contained 10 µL of 2× TB Green Premix, 0.5 µL each of forward and reverse primers (10 µM stock), 8 µL of nuclease-free water, and 1 µL of appropriately diluted cDNA. The qPCR reactions were performed on an Eppendorf Realplex2 Mastercycler (Eppendorf, Germany) using a two-step protocol with an initial denaturation at 95°C for 30 seconds, 40 cycles of denaturation at 95°C for 5 seconds, and annealing and extension for 30 seconds at 60°C. A melt curve analysis was performed after 40 cycles of qPCR. 16S rRNA was used as a normalizing internal control to calculate change in expression for all other genes. A known concentration of genomic DNA and its dilutions was used to construct a standard graph for each gene. Fold changes in gene expression for each gene were calculated with respect to the wild type using the standard graph method.

## Data Availability

All materials such as bacterial strains generated in this study will be shared upon request. Whole-genome and transcriptome sequencing data sets have been uploaded to Genbank (Projects: PRJNA996735 and PRJNA741586).

## SUPPLEMENTAL MATERIAL

File S1 mbio.02119-23-s0001.xlsx Compiled list of *mgrB* mutations in colistin-resistant *Klebsiella pneumoniae* from 17 independent studies.

File S2 mbio.02119-23-s0002.xlsx Genome sequencing results for evolution of trimethoprim resistance in *E. coli* under high and low PhoQP activity levels.

File S3 mbio.02119-23-s0003.xlsx Differential gene expression based on RNA-sequencing in Tmp^R^-A, B, and C.

File S4 mbio.02119-23-s0004.xlsx Phylogenetic distribution of PhoQ and MgrB.

Supplemental figures and captions mbio.02119-23-s0005.pdf Figures S1-S5 and figure captions to Files S1-S4.

ASM does not own the copyrights to Supplemental Material that may be linked to, or accessed through, an article. The authors have granted ASM a non-exclusive, world-wide license to publish the Supplemental Material files. Please contact the corresponding author directly for reuse.
