# Supplementary material for: Pervasive gene deregulation underlies adaptation and maladaptation in trimethoprim-resistant E. coli
Source: mBio. 2023 Nov 30;14(6):e02119-23. doi: 10.1128/mbio.02119-23 (PMC10746255; doi:10.1128/mbio.02119-23)
Supplement: Supplemental figures and captions — Figures S1-S5 and figure captions to Files S1-S4. [file mbio.02119-23-s0005.pdf]

Supplementary Material

Supplementary Figures

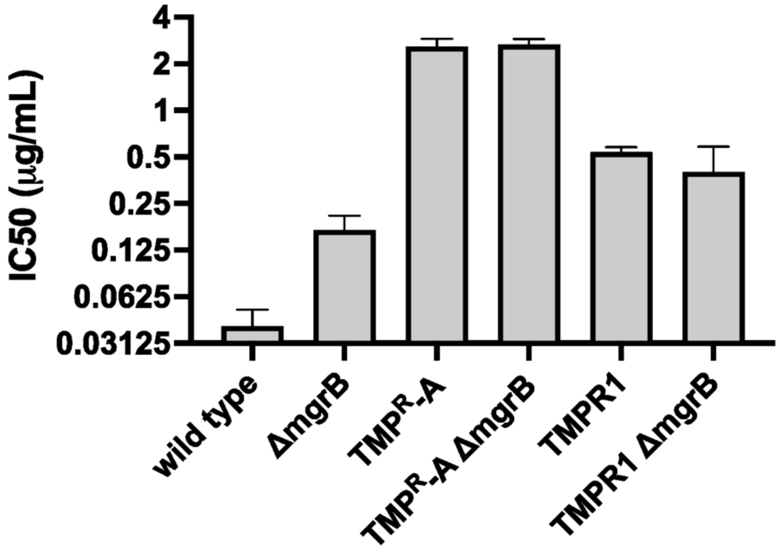

Figure S1. Replacement of trimethoprim-selected *mgrB* mutations with *ΔmgrB*::Kan allele does not impact IC50 values of resistant isolates. TMP^R-A and TMPR1 isolates had a ΔA_{98} and -23::IS1 mutations at the *mgrB* locus respectively. Mean ± S.D. from 3 independent replicates is shown.

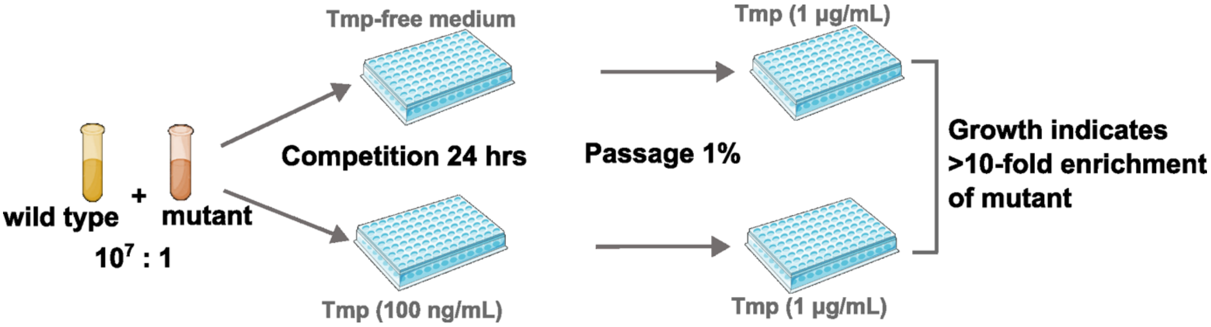

Figure S2. Experimental design to determine frequency of establishment of trimethoprim-resistant isolates over a large excess of drug-sensitive wild type *E. coli*.

Tmp^R-A

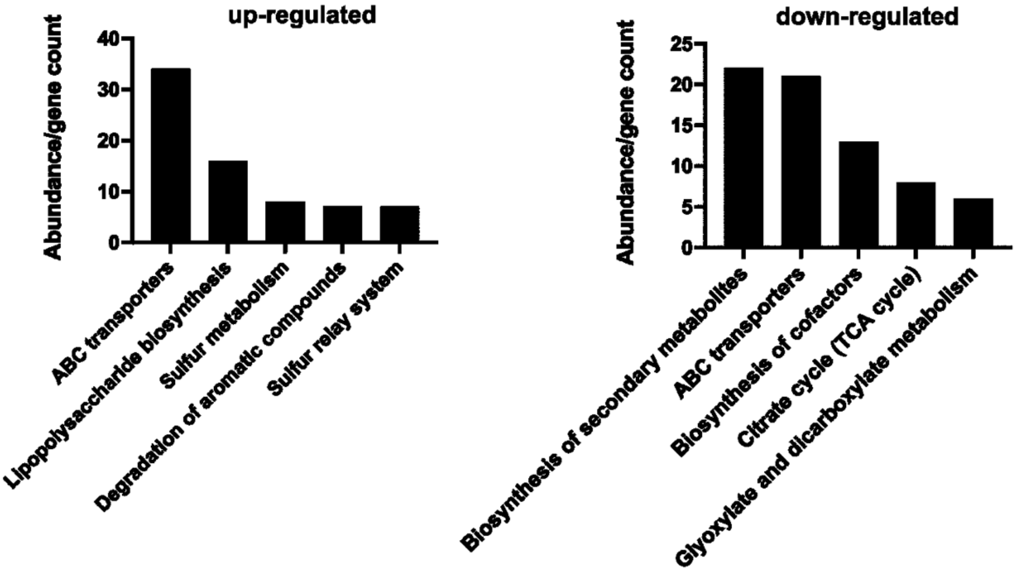

Tmp^R-B

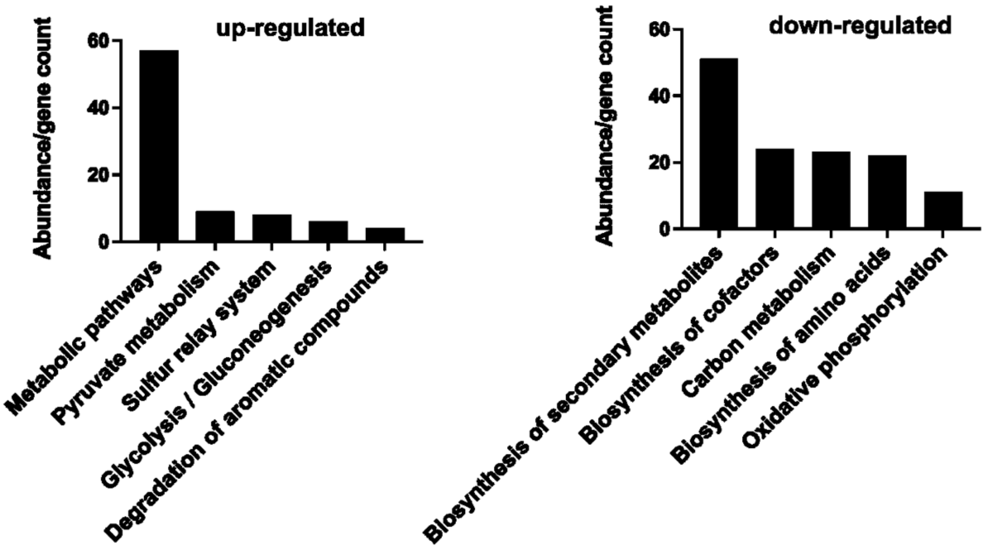

Figure S3. Pathways upregulated and downregulated in trimethoprim resistant isolates Tmp^R-A and B

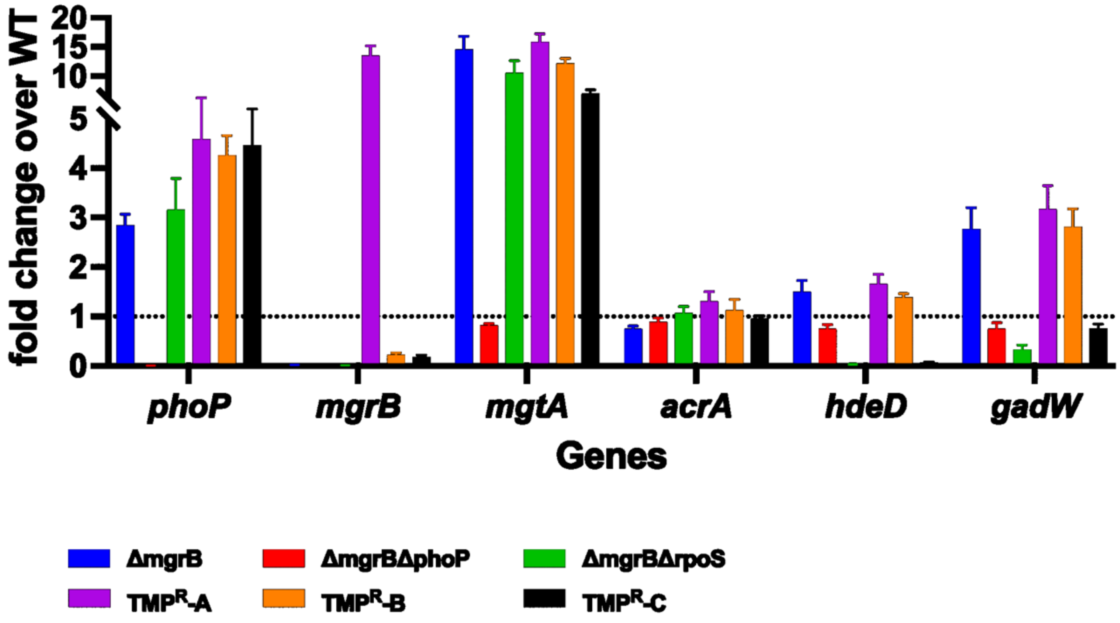

Figure S4. Quantitative real-time RT-PCR analysis of PhoP regulated genes (X-axis) in trimethoprim-resistant and genetically manipulated *E. coli* (different coloured bars). Data are expressed as fold change over wild type. Mean ± S.D. from 3 independent replicates is shown. No change (=1) is indicated by a dotted line.

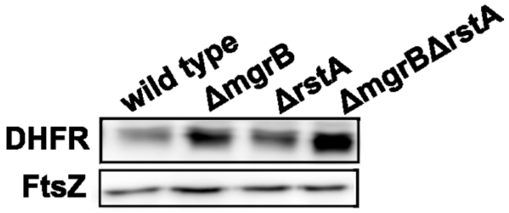

Figure S5. Expression analysis of DHFR protein in indicated mutants of *E. coli* by immunoblotting using anti-DHFR polyclonal IgG. Anti-FtsZ is used as loading control. Representative data from 3 independent replicates is shown.

Supplementary files

Supplementary file 1. Compiled list of *mgrB* mutations in colistin-resistant *Klebsiella pneumoniae* from 17 independent studies

Supplementary file 2. Genome sequencing results for evolution of trimethoprim resistance in *E. coli* under high and low PhoQP activity levels

Supplementary file 3. Differential gene expression based on RNA-sequencing in Tmp^R-A, B and C

Supplementary file 4. Phylogenetic distribution of PhoQ and MgrB
